# Preference Elicitation and Treatment Decision-Making Among Men Diagnosed With Prostate Cancer: Randomized Controlled Trial Results of Healium

**DOI:** 10.2196/46552

**Published:** 2023-10-20

**Authors:** Michael A Diefenbach, Allison Marziliano, Erin K Tagai, Halie Pfister, Emmanuel Lapitan, Simon J Hall, Manish Vira, Said Ibrahim, Kelli Aibel, Alexander Kutikov, Eric M Horwitz, Curtis Miyamoto, Adam C Reese, Suzanne M Miller

**Affiliations:** 1 Institute of Health System Science The Feinstein Institutes for Medical Research Northwell Health Manhasset, NY United States; 2 Cancer Prevention and Control Program Fox Chase Cancer Center, Temple University Health System Philadelphia, PA United States; 3 Smith Institute for Urology, Northwell Cancer Institute, Northwell Health Manhasset, NY United States; 4 Department of Urology Fox Chase Cancer Center, Temple University Health System Philadelphia, PA United States; 5 Department of Radiation Oncology, Fox Chase Cancer Center, Temple University Health System Philadelphia, PA United States; 6 Lewis Katz School of Medicine, Temple University Philadelphia, PA United States

**Keywords:** prostate cancer, decision-making, decision support, decision tool, web-based intervention, patient preferences, preference elicitation software, preference, RCT, randomized controlled trial, oncology, prostate, men’s health, emotional

## Abstract

**Background:**

Elicitation of patients’ preferences is an integral part of shared decision-making, the recommended approach for prostate cancer decision-making. Existing decision aids for this population often do not specifically focus on patients’ preferences. Healium is a brief interactive web-based decision aid that aims to elicit patients’ treatment preferences and is designed for a low health literate population.

**Objective:**

This study used a randomized controlled trial to evaluate whether Healium, designed to target preference elicitation, is as efficacious as Healing Choices, a comprehensive education and decision tool, in improving outcomes for decision-making and emotional quality of life.

**Methods:**

Patients diagnosed with localized prostate cancer who had not yet made a treatment decision were randomly assigned to the brief Healium intervention or Healing Choices, a decision aid previously developed by our group that serves as a virtual information center on prostate cancer diagnosis and treatment. Assessments were completed at baseline, 6 weeks, and 3 months post baseline, and included decisional outcomes (decisional conflict, satisfaction with decision, and preparation for decision-making), and emotional quality of life (anxiety/tension and depression), along with demographics, comorbidities, and health literacy.

**Results:**

A total of 327 individuals consented to participate in the study (171 were randomized to the Healium intervention arm and 156 were randomized to Healing Choices). The majority of the sample was non-Hispanic (272/282, 96%), White (239/314, 76%), married (251/320, 78.4%), and was on average 62.4 (SD 6.9) years old. Within both arms, there was a significant decrease in decisional conflict from baseline to 6 weeks postbaseline (Healium, *P*≤.001; Healing Choices, *P*≤.001), and a significant increase in satisfaction with one’s decision from 6 weeks to 3 months (Healium, *P*=.04; Healing Choices, *P*=.01). Within both arms, anxiety/tension (Healium, *P*=.23; Healing Choices, *P*=.27) and depression (Healium, *P*=.001; Healing Choices, *P*≤.001) decreased from baseline to 6 weeks, but only in the case of depression was the decrease statistically significant.

**Conclusions:**

Healium*,* our brief decision aid focusing on treatment preference elicitation, is as successful in reducing decisional conflict as our previously tested comprehensive decision aid, Healing Choices*,* and has the added benefit of brevity, making it the ideal tool for integration into the physician consultation and electronic medical record*.*

**Trial Registration:**

ClinicalTrials.gov NCT05800483; https://clinicaltrials.gov/study/NCT05800483

## Introduction

Shared decision-making (SDM), defined as a process in which both the patient and the health care professional work together to decide the best plan of care for the patient, is the recommended approach for prostate cancer treatment decision-making [1]. All major medical and professional organizations in the United States (eg, American Urological Association and the American Cancer Society) recommend that SDM be an essential part of patient-centered care [2]. Patients want to be involved in health decision-making, and higher quality decision-making is related to better emotional quality of life [3]. Yet, SDM is not implemented reliably in clinical practice, particularly for low-health literate patients [4,5]. Barriers to implementing SDM include lack of training in SDM protocols, lack of time, and a paternalistic attitude among providers [6].

The use of personal, technology-based decision aids empowers patients to identify and verbalize their own preferences and bring their concerns to the clinical consultation, which encourages an SDM process. Multiple studies demonstrate that the use of personal decision aids facilitates SDM [7-9]. To improve disease knowledge and facilitate SDM, our research group has developed several interactive, Internet-based decision aids for patients with prostate cancer [10-12] and those with breast cancer. The Prostate Interactive Education System (PIES) and the second-generation Healing Choices programs for prostate and breast cancer are comprehensive educational and decision tools that include several hours of text information and video-based testimonials. We demonstrated that our software enhances disease-specific knowledge, decreases decisional conflict [10,11], and increases perceived support, particularly for non-White minority patients [12]. Despite these promising results, our software programs have their limitations, particularly because they do not elicit patients’ preferences. Other limitations are that they: are not widely used in clinical practice due to the time burden for usage, lack a defined clinical pathway into the treatment consultation and SDM model, and are unlikely to be well-integrated into electronic medical records.

Our goal was to address the limitations of our prior aids (eg, risk of information overload, too time-consuming, lack of integration into physician consultation) while retaining their efficacy, and simultaneously focusing on patients’ preferences. Therefore, we reconceptualized our approach to software-guided facilitation of decision-making consistent with the SDM approach and developed Healium*.* As the scientific base on decision-making has progressed, patients’ personal preferences have been identified as central to treatment choice selection [13,14]. Patient preferences are cognitive-affective constructs, that are made from direct (ie, lived) or indirect (witnessed) experiences [15]. This combination of experiences makes preferences highly personal and powerful predictors of decision-making [16]. Despite the central role patient preferences play in SDM, they are not systematically assessed by providers, thus hindering the proper application of SDM, and limiting patients’ full understanding of their treatment choices.

Healium primarily focuses on the elicitation of treatment preferences [17], particularly for those with low health literacy who are least likely to engage in SDM. Rather than organizing the information presented by treatment modality (eg, surgery, radiation, and active surveillance), and having patients learn about each category, the newly designed program breaks down the treatment decision process from the patient’s perspective. When faced with a diagnosis of prostate cancer, the patient has unique decisional-making needs, as he must weigh the consequences of active treatment (potential for cure, negative impact on quality of life) with the consequences of active surveillance (living with uncertainty of having cancer). As such, the first decision a patient faces when diagnosed with early-stage prostate cancer is, whether they “want to be treated right now or wait” (ie, engage in active surveillance). Consequently, this is the first “gate” question patients see when accessing the Healium program. Depending on patients’ answers, they are presented with more specific information about treatment or active surveillance options that they are asked to rate in terms of acceptability (see Methods section for more details). Colorful circles that change size with increasing or decreasing acceptability provide visual feedback of the patient’s rating. After rating different options, patients are presented with a summary of their preferences identified through their rating responses and are asked to discuss those preferences with their consulting physician and explore other options in a more informed manner. The entire program can be completed in 10-13 minutes. We hypothesized that by focusing on patient preferences, patients learn what is important to them, identify questions that need clarification from their physician, and can make treatment decisions that align with their preferences and values.

Therefore, the purpose of this study is: (research question 1) to evaluate whether Healium, designed to focus on preference elicitation, is as efficacious in improving decision-making outcomes—(1) decisional conflict, (2) satisfaction with decision, and (3) preparation for decision-making, as Healing Choices in a randomized controlled trial; (research question 2) to compare the emotional quality of life—(1) anxiety/tension and (2) depression, between patients randomized to Healium and Healing Choices; and (research question 3) to assess the relationship between treatment decision and patients’ emotional quality of life—(1) anxiety/tension and (2) depression.

## Methods

### Inclusion Criteria

Patients were eligible to participate in this study if they met the following inclusion criteria: (1) they had a diagnosis of localized prostate cancer and were eligible for all treatment options (ie, surgery, radiation, active surveillance), (2) they had not yet made a treatment decision or begun treatment, and (3) they have basic proficiency (grade school level) in reading English.

### Recruitment

Study coordinators screened the electronic medical record to identify potentially eligible patients scheduled for diagnostic and treatment consultation visits. If eligible based on chart review, the study coordinator telephoned the patient, briefly introduced the study, obtained preliminary consent, and asked the patient to arrive 45 minutes prior to their upcoming appointment. On the day of the appointment, the study coordinator obtained written informed consent, implemented block randomization by the study site based on a predetermined randomization scheme, administered the baseline assessment, and was available to answer any questions or concerns. Participants completed additional assessments at 6-week and 3-month postbaseline, and were provided with US $30 in gift cards (US $10 per each of the 3 time points).

To adhere to the structure required of a randomized controlled trial and uphold scientific rigor, we administered Healing Choices as a time and attention comparison arm in the same way as Healium (ie, by research coordinators in the waiting room during visits to the clinic). However, integrating a program with several hours of information into the clinic setting, as is the case with Healing Choices, is not feasible and Healing Choices was not designed to be used as a discussion tool for SDM.

### Intervention Arm (Healium)

Participants assigned to the intervention arm completed the Healium program on a provided laptop computer in an internet-enabled clinic room. A member of the study team remained in the room to assist with technical questions but did not answer any disease or treatment-related questions. On average, it took participants 10-13 minutes to complete Healium.

Healium is a web-based platform that employs a user-centric design and aims to appeal to a low-health literate population. It features a simple language and layout, a large font size, contrasting text and background colors, a bright color palette, and the use of short labels and headings to describe content.

Healium uses plain language and easy-to-use touchscreen commands for navigation. To minimize cognitive load during decision-making, complex treatment decision-making is broken down into a series of simple gate questions that are answered in a yes or no format. The program begins by eliciting users’ preferences on whether they want to treat their prostate cancer immediately or whether they want to wait. In other words, the program offers the choice between active surveillance or active treatment (ie, surgery or radiation). If a patient chooses active surveillance, the next page contains 4 to 5 preferences (eg, side-effects or treatment features) characteristic of the selected choice. Touch or mouse controls are used to move a slider across the screen, to indicate whether the patient would be “bothered” by the selected feature (ie, range from “not at all,” to “somewhat” to “bothered a great deal”). The higher the level of “bother” the larger the corresponding-colored circle grows. The circle size serves as a visual representation of the patients’ preferences in that specific area. Once all preferences are rated and submitted, the program generates a summary report, with the different colored and sized circles included. If the patient endorses that certain symptoms associated with a particular treatment are highly bothersome (eg, no tolerance for potential urinary or sexual dysfunction), the program suggests that this particular treatment choice is not compatible with the stated preferences. The user is then prompted to revisit the prior preference rating page or to “start over” again, such as exploring a different treatment modality (eg, radiation therapy). Such an iterative process of preference elicitation and evaluation mimics a natural decision process: As patients are presented with different considerations that they are asked to rate, they might be exposed to new, important information, that might influence their treatment choice. Users are encouraged to continue to explore the tool and discuss the treatment preference summary generated by the program with their physicians.

Although not the focus of this manuscript, the development of Healium was guided by the ORBIT (Obesity-Related Behavioral Intervention Trials) model [18], which emphasizes flexible yet progressive program development steps, following prespecified clinically significant milestones, including repeating earlier phases if necessary to refine the intervention. This approach has been used successfully to guide the development of many of our web-based programs [19,20].

### Comparison Arm (Healing Choices)

Patients randomly assigned to the comparison arm received information through the Healing Choices program, accessed in the same setting and under the same conditions as patients in the intervention arm.

The Healing Choices program represents a virtual health center that patients visit to obtain disease and treatment-related information. The software was designed to be open to exploration with an intuitive layout, without restrictions in terms of order of access. Information is stored in virtual rooms, such as a library, a conference room showing videos by survivors who discuss their approach to treatment, and physician offices containing videos of physicians representing different treatment specialties. All information was extensively vetted by health education experts of the National Cancer Institute’s Cancer Information Services (CIS). See Table 1 for a head-to-head comparison of the Healing Choices and Healium programs.

**Table 1:** Comparison of Healing Choices and Healium.

	Healium	Healing Choices
Theoretical framework	Self-regulation framework	Self-regulation framework
Basic information	Included	Included
Exhaustive library	Not included	Included
Treatment decision support	Included	Included
Preference elicitation	Included	Not included
Designed for low-health literate patients	Included	Not included
Interface	Tablet based	Computer based
Time needed to review	10-13 minutes	Several hours of content

Versions of Healing Choices for prostate cancer and early-stage breast cancer were evaluated in nationwide randomized controlled trials. Analyses of Healing Choices for men with prostate cancer indicated a significant intervention effect on levels of perceived decisional support, which was greatest for non-White minority participants and patients with lower educational attainment [12]. As-treated analyses of Healing Choices for women with early-stage breast cancer showed that Healing Choices improved decision support, as well [21]. Although Healing Choices was successful in improving decisional outcomes in these trials, our goal with this manuscript is to determine whether Healium has equal success in improving decisional outcomes, while overcoming Healing Choices’ limitations (ie, time burden for usage; lack of a defined clinical pathway into the treatment consultation and SDM model, etc).

Although Healing Choices is Internet based, due to a server malfunction, 27 of the 156 (17.31%) participants randomized to the comparison arm received a paper version of Healing Choices. There were no significant differences in any demographic variables between those receiving the web version of Healing Choices and those receiving the paper version of Healing Choices (data not shown).

### Study Assessments

#### Overview

Participants completed assessments at baseline (consent), and at 6 weeks and 3 months post baseline. Areas assessed included: demographics, comorbidities, health literacy, treatment decision, decisional conflict, satisfaction with decision, preparation for decision-making, and emotional quality of life (anxiety/tension and depression).

#### Demographics

Demographic variables include age (continuous), ethnicity (Hispanic and non-Hispanic), race (White, Black or African American, Asian, Hawaiian/Pacific Islander, Other), annual income, highest level of education, employment status (employed, unemployed, or retired), marital status (single/never married, married/lives with partner, separated, divorced, widowed), and site of enrollment (Northwell Health or Fox Chase Cancer Center).

#### Comorbidities

Comorbidities were assessed with the Charlson Comorbidity Index [22], a widely used measure that is composed of a weighted index taking into account the number and severity of comorbid diseases.

#### Health Literacy

Health Literacy was assessed with the Newest Vital Sign (NVS) [23,24], a 6-item measure that determines the ability to apply health-related information to answer a series of numeracy and reasoning questions. Scores on this item range from 0 to 6, with a score of 0-1 indicating the high likelihood of low health literacy, scores from 2 to 3 indicating probable low health literacy, and a score from 4 to 6 indicating adequate literacy. When using this variable as a grouping variable in our analyses, we used a median split (median 5.0, values 0-4 indicated low health literate group and values 5-6 indicated adequate health literate group).

#### Treatment Decision

Treatment decision (surgery, radiation, and active surveillance) was assessed at 6 weeks as either surgery, radiation, active surveillance, or others.

#### Decisional Conflict

Decisional conflict was measured with the Decisional Conflict Scale (DCS) [25], a well-validated scale consisting of 16 items that assess 4 dimensions: informed, clarity, uncertainty, and support. The DCS total score is a sum of items.

#### Satisfaction With Decision

Satisfaction With Decision Scale [26] is a 9-item instrument, administered at 6 weeks and 3 months, that assesses satisfaction with medical decisions and is answered on a 5-point Likert scale (1=strongly disagree to 5=strongly agree). The total score is calculated as the sum of items.

#### Preparation for Decision-Making Scale

The Preparation for Decision-Making Scale (PDMS) [27] is a 10-item measure, answered on a 5-point scale (1=not at all to 5=a great deal) that assesses a patients’ perception of a given decision support tool’s ability to prepare a person to make a decision and to communicate with their provider. The questions touch on topics such as realizing that a decision needs to be made, thinking about pros and cons, and identifying questions for the provider. Total is the mean of items.

#### Emotional Quality of Life

Emotional quality of life (anxiety/tension and depression) was assessed using the relevant 5-item subscales of the short version of the Profile of Mood States (POMS) [28,29].

### Statistical Analyses

Analyses were conducted using SPSS (version 27; IBM). Total scores (both means and sums) were computed from individual items on continuous scales using the two-thirds rule (ie, the total was calculated if the participant answered at least 2/3 of the scale items). Means and SDs were calculated for continuous measures and frequencies and percentages for categorical variables. Independent sample 2-tailed *t* tests were used to compare 2 different groups on continuous measures. Chi-square tests were used to compare groups on categorical variables. Paired sample 2-tailed *t* tests were used to compare the change in continuous measures within one group over the course of time. One-way ANOVA was used to compare more than 2 groups on continuous measures. Two-way ANOVAs were used to evaluate the main and interaction effects of 2 categorical independent variables on a continuous dependent variable.

### Ethics Approval

This study was approved by the institutional review boards of Northwell Health (15-192) and Fox Chase Cancer Center (15-8013). The study was conducted from 2020 to 2021.

## Results

### Demographics and Clinical Characteristics

In total, 327 individuals consented to participate in the study. On average, participants were 64.2 (SD 6.9) years old. The majority of the sample was non-Hispanic (272/282, 96.45%), White (239/314, 76.11%), and had an annual household income of over US $75,000 (199/306, 65.03%). Just over half of the sample (170/318, 53.46%) obtained a bachelor’s degree or higher. About half (163/320, 50.94%) of the sample was employed and the other half (148/320, 46.25%) was retired. About 3 quarters (251/320, 78.44%) were married or living with a partner.

### Comparison Between Study Arms on Demographics

Of the 327 participants, 171 were randomized to the Healium intervention arm and 156 were randomized to the comparison arm (Healing Choices). There were no significant differences in any demographic variables (age, ethnicity, race, annual income, education level, employment status, and marital status), nor in comorbidities, health literacy, baseline anxiety/tension, or baseline depression between the 2 arms. See Table 2 for demographic information by arm.

**Table 2:** Baseline demographic variables (N=327).

Variable			Healium (n=171)		Healing Choices (n=156)		Total (N=327), n		*t* (*df*) or *χ*^2^ (*df*)			*P* value	
Age (n=320), mean (SD)			64.5 (6.79)		63.88 (7.03)		64.2 (6.9)^a^		0.81 (318)^b^			.42	
**Ethnicity (n=282), n (%)**										0.69 (1)			.41
	Hispanic	4 (1.42)		6 (2.13)		10							
	Non-Hispanic	145 (51.42)		127 (45.04)		272							
**Race (n=314), n (%)**										1.11 (4)			.89
	White	124 (39.49)		115 (36.62)		239							
	Black or African American	31 (9.87)		28 (8.92)		59							
	Asian	7 (2.23)		6 (1.91)		13							
	Hawaiian or Pacific Island	0 (0)		1 (0.32)		1							
	Other	1 (0.32)		1 (0.32)		2							
**Annual income (US $) (n=306), n (%)**										3.09 (5)			.69
	0-15,000	2 (0.65)		3 (0.98)		5							
	15,001-30,000	8 (2.61)		3 (0.98)		11							
	30,001-45,000	11 (3.59)		13 (4.25)		24							
	45,001-60,000	15 (4.90)		12 (3.92)		27							
	60,001-75,000	19 (6.21)		21 (6.86)		40							
	75,001+	106 (34.64)		93 (30.39)		199							
**Education (n=318), n (%)**										3.85 (6)			.70
	8-11 years	6 (1.89)		4 (1.26)		10							
	High school or general educational development	20 (6.29)		28 (8.81)		48							
	Vocational or tech school	9 (2.83)		7 (2.20)		16							
	Some college or university	41 (12.89)		33 (10.38)		74							
	Bachelor’s degree	45 (14.15)		35 (11.01)		80							
	Graduate degree	36 (11.32)		37 (11.64)		73							
	Doctoral degree	10 (3.14)		7 (2.20)		17							
**Employment (n=320), n (%)**										1.05 (2)			.59
	Employed	82 (25.63)		81 (25.31)		163							
	Unemployed	4 (1.25)		5 (1.56)		9							
	Retired	82 (25.63)		66 (20.63)		148							
**Marital status (n=320), n (%)**										5.35 (4)			.25
	Single or never married	15 (4.69)		11 (3.44)		26							
	Married or lives with partner	126 (39.38)		125 (39.06)		251							
	Separated	1 (0.31)		2 (0.63)		3							
	Divorced	15 (4.69)		11 (3.44)		26							
	Widowed	11 (3.44)		3 (0.94)		14							
Comorbidity (n=314), mean (SD)			2.39 (1.40)		2.41 (1.44)		2.40 (1.42)^a^		–0.10 (312)^b^			.92	
Health literacy (n=225), mean (SD)			4.41 (1.63)		4.29 (1.63)		4.35 (1.62)^a^		0.56 (223)^b^			.58	
Baseline anxiety or tension (n=310), mean (SD)			1.62 (0.45)		1.56 (0.44)		1.59 (0.44)^a^		1.24 (308)^b^			.22	
Baseline depression (n=310), mean (SD)			1.64 (0.70)		1.61 (0.69)		1.63 (0.70)^a^		0.36 (308)^b^			.72	

^a^Values here are represented in mean (SD).

^b^Values here represent results of 2-tailed *t* test.

### Comparison Between Study Sites on Demographics

Of the 327 participants, 128 were recruited from Northwell Health and 199 were recruited from Fox Chase Cancer Center. There were no significant differences between the 2 sites in age (*t*_318_=1.79, *P*=.07), level of education (*χ*^2^_6,318_=1.97, *P*=.92), marital status (*χ*^2^_4,320_=7.96, *P*=.09), annual income (χ^2^_5,306_=8.85, *P*=.12), or employment status (*χ*^2^_2,320_=3.06, *P*=.22). Ethnic (*χ*^2^_1,282_=5.14, *P*=.02) and racial distribution (*χ*^2^_4,314_=17.66, *P*=.001) differed significantly by study site. Compared to the sample from Fox Chase Cancer Center, Northwell Health’s sample had more Hispanic (30% vs 70%) and Asian (23.08% vs 76.92%) patients. Consequently, the proportion of White patients was higher at Fox Chase Cancer Center, compared to Northwell Health (67.78% vs 32.22%).

### Comparing Treatment Decision Across Study Arm and Study Site

At 6 weeks, treatment decision (surgery, radiation, and active surveillance) was assessed and compared across the study arm and study site. Treatment decision was significantly different between study arms, such that those patients randomized to Healium, compared to the comparison arm, were more likely to choose active surveillance and radiation, and less likely to choose surgery (see Table 3). There were no significant differences between the 2 study sites on treatment decisions (see Table 4).

**Table 3:** Treatment decision by arm^a^.

Variable			Healium (n=105), n (%)		Healing Choices (n=91), n (%)		Total (N=196), n		*χ*^2^ (*df*)			*P* value	
**Treatment decision**										8.42 (2)			.02
	Surgery	30 (42.3)		41 (57.75)		71							
	Radiation	48 (55.17)		39 (44.83)		87							
	Active surveillance	27 (71.05)		11 (28.95)		38							

^a^Of note, n=4 “other” were excluded (ie, seeds, cryosurgery, etc).

**Table 4:** Treatment decision by study site^a^.

Variable			Northwell Health (n=80), n (%)		Fox Chase (n=116), n (%)		Total (N=196), n		*χ*^2^ (*df*)		*P* value	
**Treatment decision**										4.45 (2)		.11
	Surgery	27 (38.03)		44 (61.97)		71						
	Radiation	42 (48.28)		45 (51.72)		87						
	Active surveillance	11 (28.95)		27 (71.05)		38						

^a^Of note, n=4 “other” were excluded (ie, seeds, cryosurgery, etc).

### Research Question 1

#### Decisional Conflict

**Figure 1:**
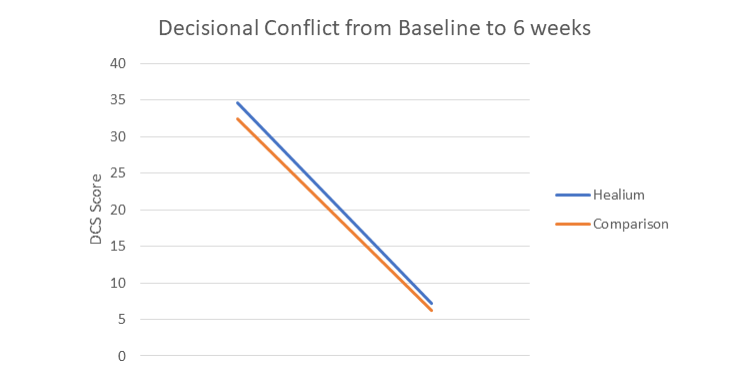
Change in decisional conflict by arm over time. DCS: Decisional Conflict Scale.

Our first research question aimed to evaluate whether Healium is as efficacious in improving decision-making outcomes, (1) decisional conflict, (2) satisfaction with decision, and (3) preparation for decision-making, as Healing Choices. Within both arms, there was a significant decrease in decisional conflict from baseline to 6 weeks post baseline. Although decisional conflict continued to decrease within both arms from 6 weeks to 3 months post baseline, the change was not significant (see Table 5 and Figure 1).

With regard to the 4 decisional conflict subscales (informed, clarity, uncertainty, support), there were significant decreases in decisional conflict on all subscales from baseline to 6 weeks post baseline within the Healium intervention arm. Informed: *t*_121_=9.53, *P*≤.001; Clarity: *t*_120_=7.4, *P*≤.001; Uncertainty: *t*_120_=9.53, *P*≤.001; and Support: *t*_122_=7.74, *P*≤.001. However, the changes for all subscales from 6 weeks to 3 months postbaseline were not significant. Informed: *t*_100_=–0.19, *P*=.85; Clarity: *t*_103_=.97, *P*=.34; Uncertainty: *t*_104_=1.92, *P*=.06; and Support: *t*_104_=1.00, *P*=.32.

Within the comparison arm, there were significant decreases in decisional conflict on all subscales from baseline to 6 weeks post baseline. Informed: *t*_98_=8.28, *P*≤.001; Clarity: *t*_99_=7.71, *P*≤.001; Uncertainty: *t*_98_=9.93, *P*≤.001; and Support: *t*_99_=6.69, *P*≤.001. Changes for subscales from 6 weeks to 3 months postbaseline were not significant except for the uncertainty subscale, which decreased significantly from 6 weeks to 3 months. Informed: *t*_90_=–0.94, *P*=.35; Clarity: *t*_90_=–0.54, *P*=.59; Uncertainty: *t*_90_=2.64, *P*=.01; and Support: *t*_88_=1.40, *P*=.16.

**Table 5:** Change in variables by arm over time.

Arm		Baseline, mean (SD)	6 weeks, mean (SD)	*t* (*df*)	*P* value^a^	6 weeks, mean (SD)	3 months, mean (SD)	*t* (*df*)	*P* value^b^
**Decisional conflict**									
	Healium	34.68 (26.99)	7.15 (15.32)	10.76 (123)	<.001; n=124	7.86 (16.42)	6.10 (14.81)	1.41 (104)	.16; n=105
	Comparison	32.45 (23.33)	6.17 (12.82)	11.49 (102)	<.001; n=103	6.18 (12.82)	5.03 (11.17)	1.08 (92)	.28; n=93
**Satisfaction with decision**									
	Healium	—^c^	—	—	—	35.19 (7.78)	36.73 (7.01)	–2.04 (102)	.04
	Comparison	—	—	—	—	35.45 (7.61)	37.52 (5.68)	–2.58 (90)	.01
**Anxiety** **or** **tension (a facet of the emotional quality of life)**									
	Healium	1.59 (0.43)	1.54 (0.41)	1.21 (116)	.23	1.53 (0.37)	1.49 (0.46)	0.75 (94)	.46
	Comparison	1.59 (0.39)	1.54 (0.42)	1.11 (102)	.27	1.54 (0.42)	1.51 (0.34)	0.49 (87)	.63
**Depression (a facet of the emotional quality of life)**									
	Healium	1.58 (0.67)	1.39 (0.51)	3.28 (116)	.001	1.37 (0.50)	1.40 (0.64)	–0.51 (94)	.61
	Comparison	1.64 (0.71)	1.42 (0.54)	3.58 (102)	<.001	1.40 (0.55)	1.33 (0.48)	1.59 (87)	.12

^a^Comparing baseline and 6 weeks.

^b^Comparing 6 weeks and 3 months.

^c^Not available.

#### Satisfaction With Decision

Within both the intervention and comparison arms, there was a significant increase in satisfaction with one’s decision from 6 weeks to 3 months (Table 5).

#### Preparation for Decision-Making

Both Healium and Healing Choices prepared patients adequately for treatment decision-making and their consultations with the physicians. At 6 weeks post baseline, there was no significant difference between Healium and Healing Choices on the preparation for Decision Making Scale (mean 3.64, SD 0.94 vs mean 3.65, SD 0.97; *P*=.90).

### Research Question 2: Emotional Quality of Life (Anxiety/Tension and Depression)

Our second research question aimed to evaluate whether Healium is as efficacious in improving emotional quality of life, (1) anxiety/tension and (2) depression, as Healing Choices. Within both the intervention and comparison arms, anxiety/tension was low at baseline and decreased minimally (but not significantly) from baseline to 6 weeks. A similar pattern was found within both arms from 6 weeks to 3 months (see Table 5).

Within both the intervention and comparison arms, there was a significant decrease in depression from baseline to 6 weeks; however, changes in depression from 6 weeks to 3 months were not significant in both arms (see Table 5).

### Research Question 3: Emotional Quality of Life (Anxiety/Tension and Depression) and Treatment Decision

Our third research question aimed to explore the relationship between treatment decisions and emotional quality of life, (1) anxiety or tension and (2) depression. There were no significant differences in anxiety or tension across treatment decisions at 6 weeks, *F*_2,185_=2.62, *P*=.08, nor at 3 months, *F*_2,158_=1.46, *P*=.24. We found a significant difference in depression at 6 weeks based on treatment decision. Surgery: mean 1.53, SD 0.64; Radiation: mean 1.32, SD 0.41; Active Surveillance: mean 1.44, SD 0.47, *F*_2,185_=3.18; *P*=.04. This difference was no longer significant at 3 months, *F*_2,158_=1.18, *P*=.31.

## Discussion

### Overview

We developed the Healium software in response to 2 primary gaps in the literature. First, it has become clear that integrating comprehensive decision and education tools with several hours of content (as is the case with Healing Choices and other similar programs) into the electronic medical record and the clinical consultation process is not feasible. Indeed, the extensive content of Healing Choices*,* consisting of physician and survivor videos, graphics, and comprehensive descriptions of treatment options, makes it impossible to use in the waiting room prior to or during patient-physician consultations. A brief tool that could be completed in a few minutes was needed to enhance the SDM experience. Second, in recent years, the decision and judgment literature has increasingly emphasized the role of patients’ preferences in treatment decision-making, especially in preference-sensitive treatment situations, as is the case with prostate cancer. Thus, an increased emphasis on eliciting patient’s preferences was needed. The Healium software program fills these two gaps: (1) it primarily focuses on the elicitation of patient preferences with regard to the 3 primary treatment options (ie, active surveillance, surgery, radiation) and (2) it can be completed by patients in 10-15 minutes, thus making it possible to integrate the program either prior to, or during, the consultation process. Last, our approach to breaking down the decision steps into a series of brief yes or no questions should make the program particularly amenable for patients with low health literacy.

This study was designed to demonstrate that the Healium software was as efficacious in reducing decisional conflict as our previously developed and tested program, Healing Choices for prostate cancer [12]. The Healing Choices program focused primarily on patient education, providing extensive information through patient and physician videos as well as written text. Eliciting patient preferences is mentioned but is not a central focus of the program. It was also not specifically designed for a low-health literate population, although information was written at a sixth-seventh grade level. Healing Choices was developed as an ancillary service to the CIS, and is intended to serve as a stand-alone program that could be used independently from physician consultations.

### Principal Results

In this study, we demonstrated that both the Healing Choices and Healium programs are successful in reducing decisional conflict. A nearly 30-point drop in decisional conflict, as achieved by both programs, is clinically relevant. Indeed, it indicates a reduction from clinically significant conflict to almost no conflict at all. The lack of decisional conflict was accompanied by an increase in decisional satisfaction from 6 weeks to 3 months postbaseline assessment and there was no difference in emotional quality of life between participants receiving the 2 programs. Remarkably, patients reported low levels of anxiety or tension at baseline, which then further declined, albeit nonsignificantly. These low levels of anxiety or tension diverge from levels published in the literature, which usually show moderate to high levels of distress after a receipt of a prostate cancer diagnosis. Depression at baseline was somewhat higher, but still at a subclinical level, and declined significantly at 6 weeks. The reason for these low levels of affect prior to the physician consultation is unclear; yet, it is possible that patients’ anxiety was lower because they were focusing on the fact that they were going to resolve their cancer threat.

Healium was successfully implemented within 2 separate clinic sites (Northwell Health and Fox Chase Cancer Center). Indeed, within each site, patients were recruited from Urology and Radiation Oncology, demonstrating the utility of using Healium before a consultation with either a surgical oncologist or radiation oncologist.

It is noteworthy that when comparing the 2 programs, patients randomized to Healium were more likely to select active surveillance than active treatment (either surgery or radiation), while those randomized to Healing Choices were more likely to select active treatment (either surgery or radiation) than active surveillance. Evidence in the literature indicates that patients who use shared decision-making, the core of Healium, tend toward choosing less aggressive treatment options, such as active surveillance. It has been suggested that the deeper processing of the pros and cons of treatment options leads to less aggressive treatment approaches.

### Implications for Future Research

This study was an initial step in a larger program of research. Next steps include evaluating the most efficient option for integration of Healium into clinical care (ie, email delivery of results to the physician vs full integration of results into the electronic medical record), audio-recording and analyzing encounters between the patients using Healium and their physicians to identify evidence of SDM as well as that patients are voicing their preferences, and examining whether patients are more likely to receive preference congruent treatment after using Healium than without Healium.

### Implications for Future Practice

Looking ahead, it is important to highlight the specific contexts and dissemination potentials of the 2 programs given their unique characteristics. First, Healing Choices’ prime objective is patient education. This is achieved by providing comprehensive disease and treatment-related information in a centralized location, in the form of written content as well as physician and survivor videos. Healing Choices is best used after a positive prostate biopsy result and a diagnosis of prostate cancer. In contrast, Healium’s advantage is its focus on patient preference elicitation and as a communication tool for the SDM process. It is most useful for the patient to complete Healium prior to the consultation with the physician. The brief completion time of Healium makes a future integration in the clinical practice feasible and graphical nature of the preference elicitation results serves as a convenient tool for starting the SDM process.

### Limitations

The study has a few limitations. First, although the minority representation was adequate, the patient population was well-educated and had a moderately high income. This makes our sample representative of patients who are more likely to seek second opinions or are visiting a comprehensive cancer center, but is less representative of the population at large. Related to this, failing to oversample African American patients was a missed opportunity, especially important given that African American men are disproportionately affected by prostate cancer with higher incidence and mortality rates. Second, our decision aid was designed for a low-health literate patient population; however, due to the ceiling effect in the health literacy variable, we could not examine whether there was a difference in our outcomes between those with low versus adequate health literacy. As such, this question will be the focus of future work, as testing among patients with very low levels of health literacy awaits.

### Conclusions

In sum, the results show that a brief treatment decision aid focusing on preference elicitation and designed for a low-health literate patient population is successful in reducing decisional conflict. Further, this brief-focused program is as efficacious as our previously tested comprehensive decision aid, Healing Choices for Prostate Cancer. Decisional satisfaction was equally high and emotional quality of life was not increased. The major advantage of Healium is its’ brevity, its utility as a discussion tool for SDM, and its promise of integration within the electronic medical record to further facilitate the treatment counseling process.

## Appendix

Multimedia Appendix 1 CONSORT-eHEALTH checklist (V 1.6.1).

## Abbreviations

CIS: Cancer Information Services
DCS: Decisional Conflict Scale
NVS: Newest Vital Sign
ORBIT: Obesity-Related Behavioral Intervention Trial
PDMS: Preparation for Decision-Making Scale
PIES: Prostate Interactive Education System
POMS: Profile Of Mood States
SDM: shared decision-making

## Data Availability

Data can be made available upon reasonable request from the author.
